# Genomic analysis of multidrug-resistant *Escherichia coli* isolated from dairy cows in Shihezi city, Xinjiang, China

**DOI:** 10.3389/fmicb.2025.1527546

**Published:** 2025-02-26

**Authors:** Abdullahi Bello, Siqi Ning, Qi Zhang, Wei Ni, Shengwei Hu

**Affiliations:** Department of Biology, College of Life Sciences, Shihezi University, Shihezi City, China

**Keywords:** *E. coli*, AMR, MDR, ESBL, MGE, genomes, genes

## Abstract

**Introduction:**

Dairy farming plays a vital role in agriculture and nutrition; however, the emergence of antimicrobial resistance (AMR) among bacterial pathogens poses significant risks to public health and animal welfare. Multidrug-resistant (MDR) *Escherichia coli* strains are of particular concern due to their potential for zoonotic transmission and resistance to multiple antibiotics. In this study, we investigated the prevalence of AMR and analyzed the genomes of two MDR *E. coli* isolated from dairy cows in Shihezi City.

**Methods:**

Fecal samples were collected from dairy cows, and *E. coli* strains were isolated. Antibiotic susceptibility testing was conducted using the Kirby-Bauer disk diffusion method against 14 antibiotics. Two MDR isolates (E.coli_30 and E.coli_45) were selected for whole-genome sequencing and comparative genomic analysis. The Comprehensive Antibiotic Resistance Database (CARD) was used to identify AMR genes, and virulence factors were analyzed. Phylogenetic analysis was performed to determine the evolutionary relationships of the isolates, and a pangenome analysis of 50 *E. coli* strains was conducted to assess genetic diversity. The presence of mobile genetic elements (MGEs), including insertion sequences (IS) and transposons, was also examined.

**Results:**

Among the *E. coli* isolates, 22.9% exhibited MDR, with high resistance to imipenem and ciprofloxacin, while gentamicin and tetracycline remained the most effective antibiotics. Genomic analysis revealed key AMR genes, including *mphA*, *qnrS1*, and *bla*_CTX-M-55_ (the latter found only in E.coli_45), conferring resistance to macrolides, quinolones, and beta-lactams, respectively. Virulence genes encoding type III secretion systems (TTSS) and adhesion factors were identified, indicating pathogenic potential. Phylogenetic analysis showed that E.coli_30 and E.coli_45 originated from distinct ancestral lineages. The presence of two extended-spectrum β-lactamase (ESBL) genes in E.coli_45 was noticeable, so we studied their global and national distribution using evolutionary analysis. We found that they are endemic in *E. coli*, Salmonella enterica, and Klebsiella pneumoniae. Pangenome analysis revealed significant genetic diversity among *E. coli* strains, with unique genes related to metabolism and stress response. This indicates the bacteria’s adaptation to various environments. MGEs were identified as key contributors to genetic variability and adaptation.

**Discussion:**

This study highlights the growing threat of MDR *E. coli* in dairy farms, emphasizing the critical role of MGEs in the spread of resistance genes. The genetic diversity observed suggests strong adaptive capabilities, justifying the need for continuous AMR surveillance in livestock. Effective monitoring and mitigation strategies are essential to prevent the dissemination of MDR bacteria, thereby protecting both animal and public health.

## 1 Introduction

Dairy cows provide indispensable benefits to society, as they contribute to several sectors such as agriculture, nutrition, social aspect of, the economy, and the environment. Economically, dairy cows are essential because they are the largest contributors to the dairy industry as major milk producers, thus generating employment to people and supporting trade (Cabrera et al., 2008). They contribute to our nutrient intake as their milk and dairy products are rich in proteins, calcium, and vitamins (Islam et al., 2019). In agriculture, they contribute to sustainable farming through manure production and consumption of residual crops (Rotz et al., 2011). Dairy farming supports rural communities around the globe and it holds cultural significance for many people (Neethirajan, 2023). The industry is also vital to our environment, as a well-managed dairy farm can help mitigate climate change (Rotz et al., 2020).

However, alongside the benefits of dairy farming, there are several health risks posed by zoonotic pathogens. *E. coli* was first discovered in 1885 by Theodor Escherich and is the most extensively studied bacterium in the field of microbiology (Pakbin et al., 2021). This bacterium is a common inhabitant of the gastrointestinal tract of humans and animals, and it has been increasingly identified as a carrier of AMR genes (Yasir et al., 2020). AMR genes allow microorganisms to withstand the effects of antibiotic that once killed them or inhibit their growth. They are a significant concern in public health, veterinary medicine, and agriculture (Abdelfattah et al., 2021). A specific concern is MDR, where bacteria develop resistance to multiple antibiotic classes, and this causes difficulty in treatment.

One of the pressing issues in dairy facilities today is the interplay between drug use and drug resistance (Romeo et al., 2023; Murray et al., 2022). Bacteria that develop resistance to antibiotics have led to the inability to treat some infections. In 2019, the World Health Organization reported nearly a million deaths due to AMR, with projections suggesting that this number could rise to 20 million and incur costs that would exceed a trillion dollars if the current trends persist (Uddin et al., 2021; Watkins and Bonomo, 2016).

In addition to AMR, some *E. coli* pathotypes are known to be pathogenic (Liu et al., 2021) and they are capable of causing infections such as diarrhea in both humans and farm animals (Ntuli et al., 2016). Pakbin et al. (2021) discussed the virulence genes of some *E. coli* strains that are the leading causes of several stomach and urinary tract diseases. They also discussed how those strains cause infections by destabilizing the functions of their host cells through their virulence factors (Pakbin et al., 2021).

To address the aforementioned problems and curb AMR spread in both animals and humans, it is vital to study and monitor the emergence of AMR in our societies. In this study, we explored the phenotypic patterns of antibiotic resistance in *E. coli* strains isolated from the intestinal tracts of dairy cows. Additionally, we conducted a detailed genomic analysis of two MDR *E. coli* strains and investigated AMR genes and virulence factors, including their mechanisms. We also examined *E. coli* diversity using a pangenome analysis and explored the role of MGEs in the spread of AMR genes and *E. coli* diversity.

## 2 Materials and methods

### 2.1 Study area and sample collection

A cross-sectional study was conducted in Shihezi City located in the Xinjiang Autonomous Region of China. The study focused on two primary locations: the Shihezi University dairy farm that specializes in breeding dairy cows using advanced technologies and a privately owned dairy farm on the outskirts of the city. Shihezi features a semi-arid climate with extreme seasonal temperatures and fertile plains, and it relies on river irrigation that provides favorable conditions for dairy farming (Yang et al., 2021). The study population consisted exclusively of dairy cows, chosen for the isolation of *E. coli* from their intestinal tracts to investigate the prevalence and characteristics of the bacteria. Fresh fecal samples were collected immediately after excretion from the middle of the fecal matter to prevent environmental contamination. Samples were placed in 50-ml centrifuge tubes, chilled on ice for preservation, and transported to the laboratory for immediate processing (Lupindu, 2017).

### 2.2 Bacterial isolation and identification

*Escherichia coli* was isolated from fecal matter using a modified protocol based on the guidelines of Alcock (Ju and Willing, 2018). The isolation was performed via culture techniques on MacConkey, Eosin Methylene Blue, and Luria Bertani agar using the streaking method. Pure isolates were obtained using subculturing that was repeated three times. Colony PCR was then employed for the molecular identification of the 16S rRNA gene using universal primers 27F and 1492R to amplify all nine variable regions (Lane et al., 1985). The PCR was conducted in a 25-μl volume under specific thermal cycling conditions that included an initial denaturation at 95°C followed by 35 cycles of denaturation, annealing, and extension. Gel electrophoresis was performed to confirm the amplification and integrity of the 16S rRNA gene. After validation, 16S rRNA gene sequencing was performed. Raw sequence data were processed using BioEdit (Hall, 1999) and Trimmomatic (Bolger et al., 2014) to remove low-quality reads. This was followed by sequence alignment and analysis in MEGA11 (Tamura et al., 2021) to identify single nucleotide polymorphisms (SNPs), conserved sites, and variable regions, facilitating the identification of the target bacteria.

### 2.3 Drug resistance test

Based on published data regarding drugs to which *E. coli* is known to be susceptible (Matuschek et al., 2014; Hombach et al., 2013), we selected 14 different antibiotics for testing. Antimicrobial susceptibility testing was conducted using the Kirby-Bauer disk diffusion method, which involves placing a 6-mm filter paper disk impregnated with a specific amount of antibiotic on a culture plate pre-inoculated with a bacterial suspension (Hudzicki, 2009). Bacterial strains were initially prepared in suspensions, and the optical density was checked at 625 nm to achieve a 0.5 McFarland standard (Benkova et al., 2020). Absorbance values within the range of 0.08–0.13 were considered acceptable according to Hudzicki’s guidelines (Hudzicki, 2009).

After incubation, the diameters of the zones of inhibition around each antimicrobial disk were measured using a ruler and recorded in millimeters. These measurements were used to classify each isolate as susceptible, intermediate, or resistant to the antibiotics tested (Matuschek et al., 2014; Hombach et al., 2013).

### 2.4 Data analysis

Python scripts were used to analyze the data generated from the antimicrobial tests. The Python libraries utilized in this research included Pandas for data manipulation (McKinney, 2010); NumPy for numerical computation and array operations (Harris et al., 2020); Matplotlib for creating static, interactive, and animated visualizations (Hunter, 2007); and Seaborn for its high-level interface for drawing good and informative statistical graphics (Waskom, 2021).

### 2.5 Library preparation and genome sequencing

Two multidrug-resistant isolates, designated as E.coli_30 and E.coli_45, were selected for genome sequencing. These two strains were chosen based on their distinctive resistance patterns observed during the susceptibility testing. E.coli_30 demonstrated resistance to all tested antibiotics except aztreonam, while E.coli_45 was resistant to all antibiotics except amikacin. This added layer of resistance compared with the other MDR isolates highlights their unique resistance profiles. This made them ideal candidates for whole-genome sequencing (WGS) to further investigate the genetic mechanisms underlying their extreme drug resistance.

DNA samples were prepared for sequencing by generating indexed libraries for each isolate. The genomic DNA of the two MDR *E. coli* was fragmented using sonication to an average size of 350 base pairs (bp). The fragmented DNA underwent a series of modifications, including end repair, A-tailing, and ligation with Illumina full-length adapters. This was followed by PCR amplification and purification of the resulting products. The library quality was assessed using qPCR to ensure integrity and concentration. Libraries that met the quality standards were pooled according to their effective concentrations and the desired data output. Sequencing was then performed on an Illumina platform using a paired-end 150 bp (PE150) strategy (Caporaso et al., 2012).

### 2.6 Data processing

Quality control of raw sequencing data was conducted using FastQC v0.12.1 (Andrews, 2010) followed by *de novo* genome assembly using SPAdes genome assembler v3.15.5 (Bankevich et al., 2012). To detect and assemble plasmid DNA, PlasmidSpades pipeline was used, and plasmidFinder-2.1.6 (Carattoli et al., 2014) was employed to confirm the presence of known plasmid replicon sequences in the genomes. The assembled genomes were further evaluated using QUAST v5.2.0 (Gurevich et al., 2013), to ensure the selection of high-quality sequences for downstream analysis. Gene annotations were performed using Prokka v1.14.6 (Seemann, 2014) to identify coding sequences and gene structures. A graphical map of the two genomes and the plasmid was generated using nucleotide sequences of the annotated contigs (fna file) in fasta format. This was achieved using one of the CARD web tools at https://proksee.ca/tools/card (Alcock et al., 2023).

### 2.7 Identification of resistance genes and virulence factors

To identify and characterize antimicrobial resistance genes and associated resistance mechanisms, we utilized the Resistance Gene Identifier (RGI) tool v6.0.3 (Alcock et al., 2023; Jia et al., 2016), which analyzes the sequences against the CARD database (Alcock et al., 2020). To effectively visualize and interpret the data, we generated RGI wheels by uploading the RGI results with json extension (JSON file) to the CARD website (Jia et al., 2016).^1^ To identify and characterize the virulence genes, we used Abricate 1.0.1 (Seemann, 2024a), which analyzed the sequences against the Virulence Factor Database (VFDB) (Chen et al., 2016).

### 2.8 Evolutionary studies

#### 2.8.1 Phylogenetic and pangenome analyses

Evolutionary and pangenome analyses were performed to examine *E. coli* diversity. Several *E. coli* genomes were downloaded from the National Center for Biotechnology Information (NCBI) database, and 17 unpublished genomes were obtained from a colleague for multilocus sequence typing (MLST). The MLST analysis was performed using mlst 2.23.0 (Seemann, 2024b) to identify the sequence types (STs) of all the genomes intended for the phylogenetic studies and pangenome analysis. The objective was to select genomes with different STs to explore the diversity between our two isolates and other *E. coli* strains. The typing scheme used was the Achtman scheme, version 4 (ecoli_achtman_4), which is one of the standard MLST schemes for *E. coli*. All genomes selected from the MLST for evolutionary studies were annotated using prokka to generate general feature format files that are suitable for pangenome studies.

Pangenome analysis was conducted using rapid large-scale prokaryote pangenome analysis (roary 3.13.0) (Page et al., 2015). The analysis generate core genome alignments (Katoh and Standley, 2013), which were used to infer genome phylogeny based on conserved genes across all genomes selected through MLST analysis. An evolutionary tree was constructed using FastTree 2.1.11 (Price et al., 2010) that efficiently handled the large dataset and produced the phylogenetic tree. Additionally, pangenome visualizations, including pie chart, histogram, and matrix, were generated using a Python script developed by roary (Page et al., 2015).

A phylogenetic analysis was conducted for the plasmid identified in E.coli_30. The analysis was based on nucleotide similarity, as a BLASTN 2.16.0+ (Zhang et al., 2000) search was performed to identify plasmid DNA similar to our plasmid (Plasmid30). The top 50 plasmid genomes from the blast search were selected, and phylogeny was inferred using FastTree based on their core genomes. All phylogenetic trees from this study were visualized, analyzed, and interpreted using the Interactive Tree of Life: iTOL: Upload a new tree (embl.de) (Letunic and Bork, 2021).

#### 2.8.2 Study of extended-spectrum beta-lactamase genes

Phylogenetic analysis was conducted for the ESBL genes, *bla*_TEM-1_ and *bla*_CTX-M-55_, identified in E.coli_45 genome. One phylogenetic tree was inferred using the protein sequences of these genes via an NCBI Blastp search, while another phylogeny for the two genes focused specifically on the endemic strains from China. *E. coli* genomes submitted to NCBI in the past 5 years were downloaded, and the two ESBL genes were extracted from these genomes. Multiple sequence alignment was performed using MUSCLE (Edgar, 2004), and phylogeny was inferred using IQ-TREE multicore version 2.3.0 (Minh et al., 2020; Nguyen et al., 2015). The analysis included ModelFinder selection (Kalyaanamoorthy et al., 2017), tree reconstruction, and ultrafast bootstrap with 1,000 replicates (Hoang et al., 2018). Phylogenetic relationships were inferred using Maximum Likelihood (ML), with bootstrap replication set at 1000. Evolutionary distances were estimated using ML and presented in units of base substitutions per site (scale 0.01). Default settings were used for all other parameters.

### 2.9 Mobile genetic elements

In addition to the plasmid identified in one of the genomes, other mobile genetic elements, such as IS, prophage regions, and more, were investigated using MGEfinder (Durrant et al., 2020), MobileOG-db (Brown et al., 2022), Virsorter (Roux et al., 2015), Alien Hunter (Vernikos and Parkhill, 2006), PhiGARo (Starikova et al., 2020), and ISfinder (Siguier et al., 2006). This investigation was essential, as many AMR and virulence genes are disseminated via mobile elements. Their presence and roles helped to explain the significant genetic diversity observed between our isolates.

## 3 Results

### 3.1 Phenotypic analysis of antibiotic resistance

**Figure 1:**
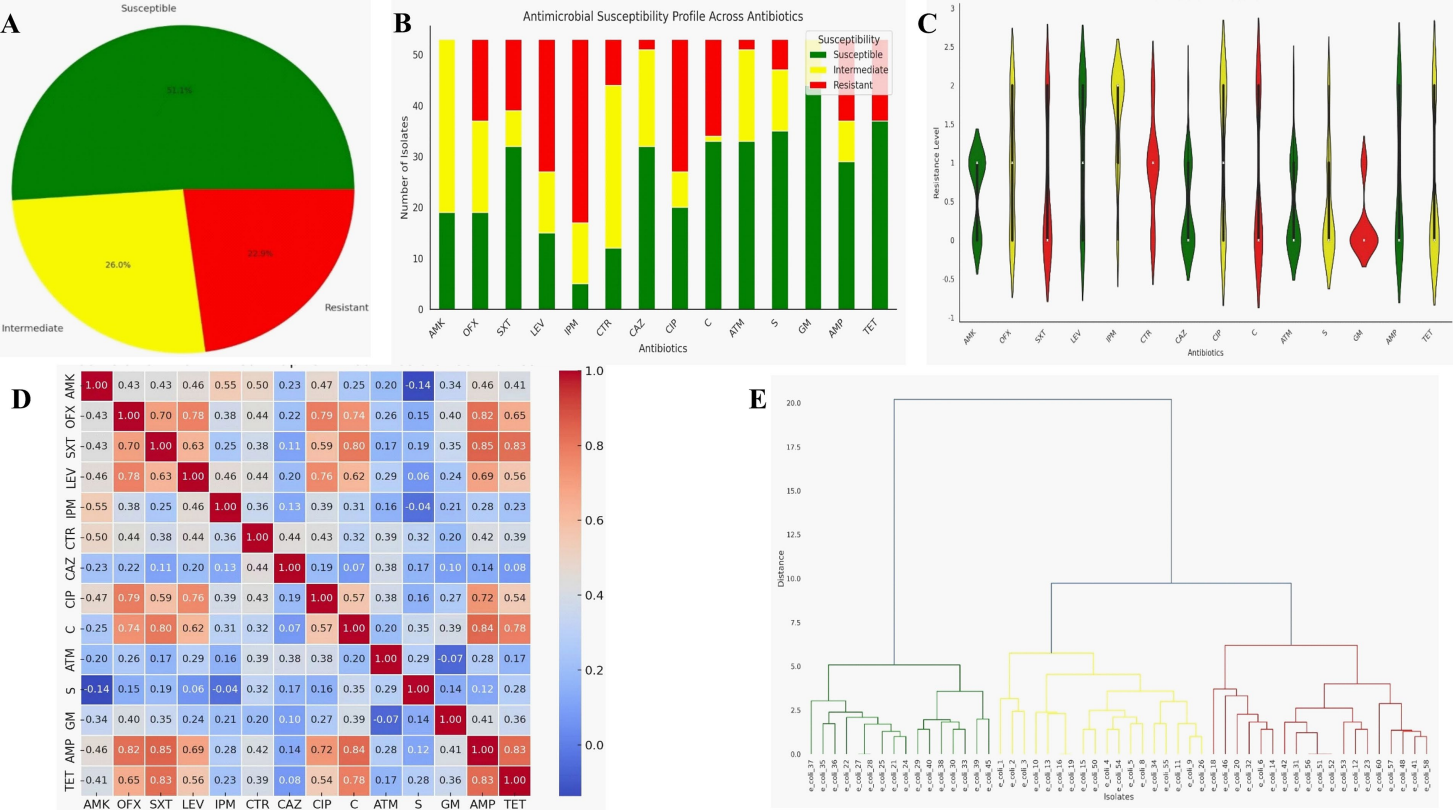
Phenotypic analysis of antibiotic resistance in *E. coli* isolates. AMK, amikacin; OFX, ofloxacin; SXT, trimethoprim/sulfamethoxazole; LEV, levofloxacin;IPM, imipenem; CTR, ceftriazone; CAZ, ceftazidime; CIP, ciprofloxacin; C, chloramphenicol; ATM, aztreonam; S, streptomycin; GM, gentamicin; AMP, ampicillin; TET, tetracycline. **(A)** Proportion of isolates’ response across all antibiotics. **(B)** Antibiotics response profile for tested isolates. **(C)** Violin plot showing the distribution and density of resistance level across 14 antibiotics for all tested *E. coli* isolates. **(D)** Correlation matrix of antibiotics based on resistance phenotype. Values closer to 1 on the heatmap indicate strong positive correlation, while values closer to 0 suggest little to no correlation. **(E)** Hierarchical cluster analysis of *E. coli* isolates based on their median response to the antibiotics tested. The green, yellow, and red clusters represent resistant, intermediate, and susceptible isolates, respectively.

The susceptibility test revealed that 51.1% of the isolates were susceptible to the tested antibiotics, 26% showed an intermediate response, and 22.9% were resistant (Figure 1A). By direct count, 26 out of the 53 isolates tested were multidrug-resistant. The highest level of resistance was observed against imipenem (IMP) (60%). In contrast, amikacin (AMK), ofloxacin (OFX), levofloxacin (LEV), ceftriaxone (CTR), and ciprofloxacin (CIP) demonstrated a more balanced distribution across susceptibility, intermediate, and resistance categories. Trimethoprim/sulfamethoxazole (SXT), ceftazidime (CAZ), chloramphenicol (C), aztreonam (ATM), streptomycin (S), gentamicin (GM), ampicillin (AMP), and tetracycline (TET) were the most effective drugs, with GM and TET showing the highest efficacy against over 65% of the *E. coli* isolates (Figures 1B,C).

Furthermore, we calculated the correlation coefficients for all the antibiotics based on the isolates’ responses to assess potential relationships between them. Strong positive correlations were observed in the resistance profiles between certain antibiotics, such as STX and AMP, C and AMP, and TET and STX, with correlation coefficients of 0.85, 0.84, and 0.83, respectively (Figure 1D). These strong positive correlations suggest that isolates resistant to one antibiotic are often resistant to the other, possibly due to similar resistance mechanisms, such as those found within the same antibiotic class. Conversely, some antibiotics, such as GM and LEV, ATM and TET, and CAZ and TET, showed little to no correlation, indicating distinct mechanisms of action (MOA) or resistance. In some cases, we also observed negative correlations, suggesting that the isolates exhibited different MOA.

Finally, we clustered the isolates based on their median score/response to antibiotics and identified distinct response patterns (Figure 1E). Nineteen strains were classified as susceptible, 18 as intermediate, and 16 as resistant. A key observation was that isolates in the first cluster (resistant strains) exhibited resistance to multiple antibiotics. This result indicates that these strains may require special attention to manage the spread of resistance effectively. Additionally, some isolates classified as MDR negative or positive appeared to have intermediate responses due to their median scores. Finally, we also observed that some MDR isolates appeared in the susceptible category. This was because, despite being MDR, they were susceptible to most of the antibiotics tested and this resulted in a susceptible median score.

### 3.2 Genome assembly and annotations

**Figure 2:**
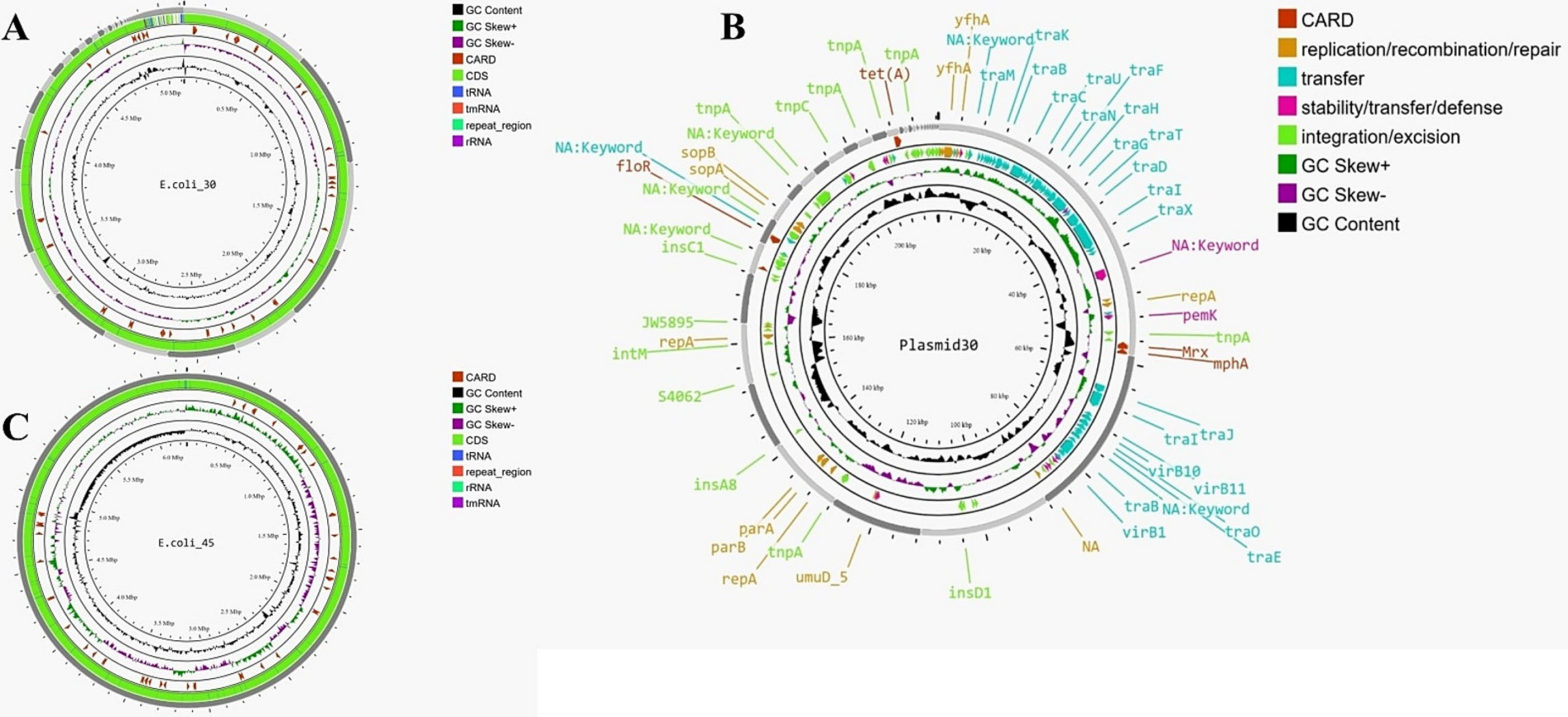
**(A)** Genome map of E.coli_30. **(B)** Genome map of E.coli_45. **(C)** Circular map of plasmid30 (plasmid found in E.coli_30).

E.coli_30 and E.coli_45 were selected for genome sequencing after susceptibility testing, as they demonstrated resistance to most of the antibiotics tested, in contrast to the other isolates. After sequencing, *de novo* assembly of E.coli_30 (Figure 2A) revealed the number of scaffolds (75) was slightly lower than the number of contigs (84). The N50 value was significantly higher for scaffolds (344,767 bp vs. 246,478 bp), indicating better assembly continuity. The total lengths of contigs and scaffolds are similar, at approximately 5.12 million bp, with an identical GC content of 50.92%. However, the scaffolding process introduced gaps, as indicated by an increase in ‘N’s per 100 kbp (17.8 in scaffolds compared to 0.02 in contigs). Annotation of this genome revealed 4,767 genes, including 4,663 coding sequences (CDS), eight rRNA genes, 95 tRNA genes, three repeat regions, and one tmRNA.

For the plasmid identified in E.coli_30 (Plasmid30), the total length of the assembly was approximately 218,143 bp, with the largest contig measuring 58,599 bp. The N50 value was 23,145 bp, while the N90 value was 3,131 bp for contigs and 3,150 bp for scaffolds. The GC content remained consistent at 50.58%, and no mismatches were found in the contigs. In contrast, the scaffolds exhibited 47.05 ‘N’s per 100 kbp and a total of 100 ‘N’s, indicating gaps introduced during the scaffolding process. In general, we can say that the scaffolding process slightly reduced the number of contigs, maintained the assembly’s length and continuity, and introduced a few gaps. Figure 2B shows the map of this plasmid, and it highlights various genes and elements that contribute to its functionality and antibiotic resistance.

The second genome, depicted in Figure 2C, had a total assembly length of approximately 4.99 million bp, with a GC content of 50.57%. The number of scaffolds (176) was slightly lower than the number of contigs (185), and the N50 value was higher for scaffolds (151,645 bp vs. 150,606 bp), indicating better assembly continuity. However, the scaffolding process introduced gaps, as reflected by the significant increase in ‘N’s per 100 kbp (15.83 in scaffolds compared to 0.02 in contigs). Overall, the scaffolding effectively linked contigs into longer sequences and enhanced the assembly’s utility for downstream analyses despite the expected introduction of gaps. Annotation of the scaffolds identified 5,076 genes (several were hypothetical proteins), including 4,953 CDS, eight rRNA genes, 114 tRNA genes, four repeat regions, and one tmRNA.

### 3.3 Plasmid replicon sequences

PlasmidFinder-2.1.6 (Carattoli et al., 2014) was used to confirm the presence of known plasmid replicon sequences within the plasmid genome identified in E.coli_30. Four plasmid replicon sequences were detected: *Col440I* with 94.74% identity over 114 bp in the 14th locus, IncFIB (*AP001918*) with 98.39% identity over 682 bp in the 7th contig, IncFII (*pCoo*) with 96.56% identity over 262 bp in the first contig (contig node 1), and *p0111* with 98.64% identity over 885 bp in contig node 5. The presence of these plasmid replicon sequences confirmed that E.coli_30 harbors plasmid within its genome. No hits were detected for plasmid types Inc18, NT_Rep, Rep1, Rep2, Rep3, RepA_N, RepL, and Rep_trans, indicating the absence of these plasmid types in the isolate. A nucleotide BLAST search was conducted to identify the origins of these replicons and the result revealed that all the sequences were from *E. coli*, except for *Col440I*, which originated from *Klebsiella pneumoniae*.

### 3.4 Antimicrobial resistance ontology

**Figure 3:**
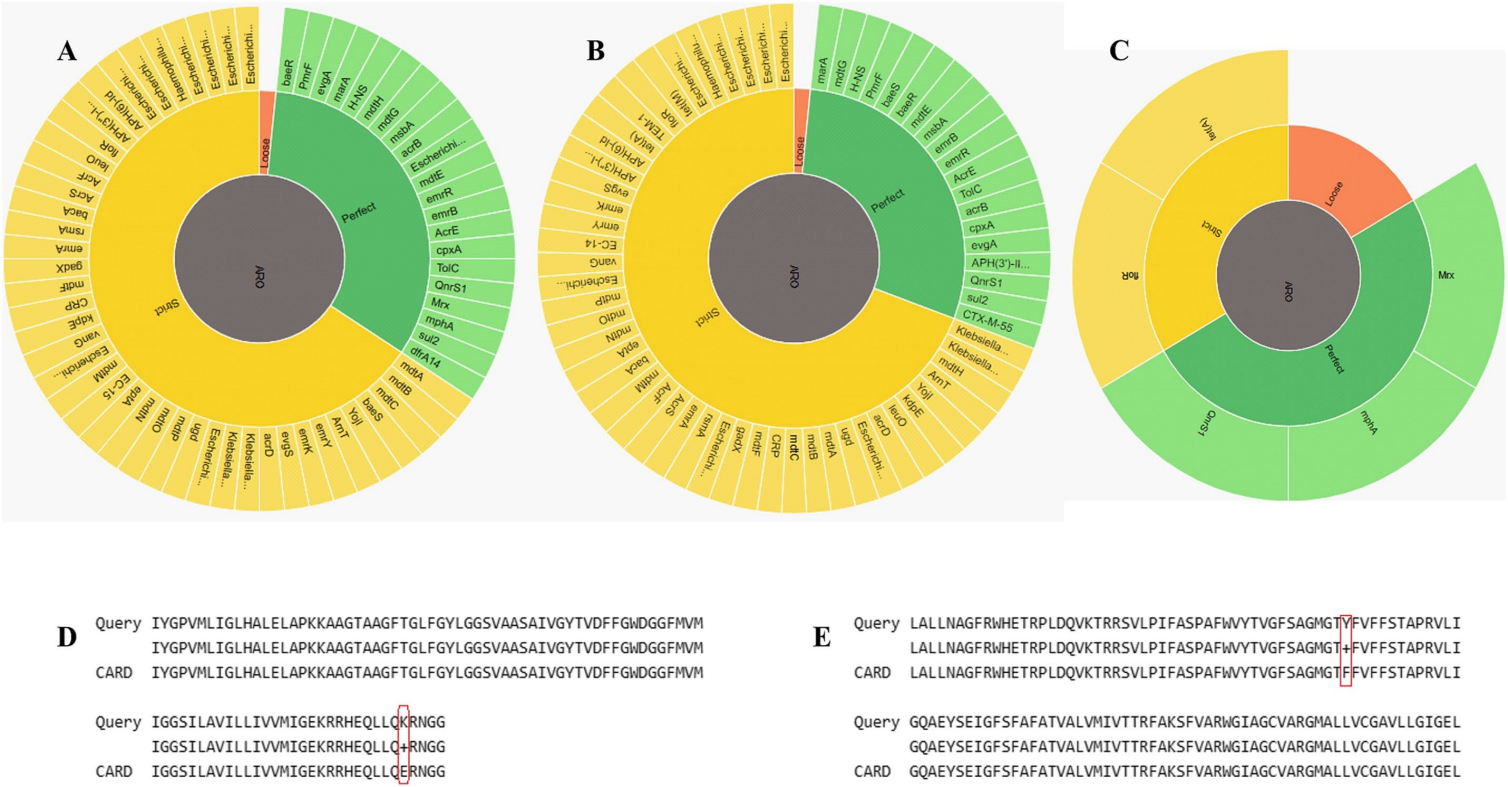
Identification of AMR genes. **(A)** RGI wheel of AMR genes identified in E.coli_30. **(B)** RGI wheel of AMR genes in E.coli_45. **(C)** RGI wheel of AMR genes in Plasmid30. **(D)** Partial sequence alignment of E.coli_30 GlpT protein showing E448K mutation. **(E)** Sequence alignment showing amino acid SNP in the *floR* gene of plasmid30.

RGI CARD investigations of the two genomes revealed the presence of multiple AMR genes (Figure 3), each contributing to antibiotic resistance through various mechanisms. Both *E. coli* genomes exhibited MDR, but the plasmid in E.coli_30 (Plasmid30) adds a layer of complexity to its resistance mechanisms. While both genomes rely heavily on efflux pumps as a primary resistance mechanism, E.coli_30 was found to have additional resistance factors from its plasmid.

Both genomes contained resistance-nodulation-cell division (RND) efflux pump systems (*acrA*, *acrB*, *AcrF*, *mdtA*, *mdtB*, and *mdtC*) that confer resistance to multiple classes of antibiotics, including fluoroquinolones, cephalosporins, tetracyclines, and macrolides. The major facilitator superfamily (MFS) pumps (e.g., *emrK*, *emrY*, *mdfA*, and *mdtM*) in both genomes offer resistance to tetracyclines, phenicols, and other antibiotics. The genomes also include ATP-binding cassette (ABC) transporters like *msbA* and *TolC* that contribute to resistance to nitroimidazoles and peptide antibiotics. Plasmid30 significantly contributes to E.coli_30 resistance, particularly to macrolides, phenicols, and fluoroquinolones (Figure 3C). It carries macrolide phosphotransferase resistance gene (*mphA*) that is not found in E.coli_45 and is involved in antibiotic inactivation.

Although both genomes had MDR profiles, E.coli_45 displayed a broader range of efflux pump systems and additional resistance mechanisms (Table 1), such as protein overexpression and regulatory mutations (e.g., *soxR*, *soxS*, and *marA*). These mutations enhance antibiotic efflux and reduce β-lactam permeability. Moreover, E.coli_45 includes more entries related to aminoglycoside resistance through inactivation mechanisms (e.g., *APH(3′)-IIa,* and *APH(3″)-Ib*). Supplementary Tables S1, S2 summarize the detailed RGI findings for both genomes.

**Table 1:** Similarities and differences between E.coli_30 and E.coli_45 RGI findings.

Feature	E.coli_30	E.coli_45
Efflux pumps	RND, MFS, SMR, ABC, plasmid-borne efflux	RND, MFS, SMR, ABC, broader diversity
Antibiotic target alteration	Quinolone target protection (plasmid)	More mutations (*soxR*, *marR*), broader range
Antibiotic inactivation	Plasmid-borne macrolide inactivation	More aminoglycoside inactivation mechanisms
Plasmid	Present; it contributes to MDR	No plasmid detected
Aminoglycoside resistance	Limited inactivation mechanisms	Multiple aminoglycoside-modifying enzymes
β-Lactam resistance	Absent	TEM-1, CTX-M-55

### 3.5 Virulence associated genes

Abricate analysis of the two genomes revealed not only similarities in their pathogenic mechanisms; but also some differences in specific gene clusters (Supplementary Tables S3, S4). Both E.coli_30 and E.coli_45 rely on several core virulence factors, particularly in adhesion, nutrient acquisition, and secretion systems. For instance, both strains possess genes involved in type 1 fimbriae (*fimA*, *fimB*, and *fimC*), *E. coli* common pilus (*ykgK/ecpR* and *yagZ/ecpA*), and colonization factor antigens. Both strains also possess the curli fiber system (*csgA*, *csgB*, and *csgC*) that is important for biofilm formation. They share similar enterobactin siderophore systems for iron acquisition, with genes such as *entA*, *entB*, *entC*, *fepB*, *fepC*, *fepD*, *fepE*, and *fepG*. However, the salmochelin siderophore system was found only in E.coli_30 and its plasmid, giving it an advantage in nutrient acquisition over E.coli_45.

Type III secretion systems (TTSS) were detected in both genomes, with genes such as *espX1, espX4*, and *espX5* encoding effector proteins that manipulate host cell signaling and immune defenses. However, E.coli_45 showed a slightly broader range of TTSS-related genes, such as *espL1* and *espR1*, indicating a wider repertoire of effector proteins. Furthermore, we detected a type VI secretion system (T6SS) in E.coli_45, with genes such as *tssA* and *hcp1/tssD1* that were absent in E.coli_30. T6SS gives E.coli_45 an advantage in bacterial competition and host interaction by enabling it to deliver toxic effectors to other bacteria or host cells.

Both strains relied on the enterobactin siderophore system for iron/nutrient acquisition. However, E.coli_45 was found to have additional enterobactin genes, such as *entS*, *fepD*, and *fepE*, making its siderophore system potentially more efficient than that of E.coli_30. We also identified an unknown protein related to the TraJ family that is encoded by AAA92657 in E.coli_45 and is involved in the regulation of invasin.

### 3.6 Evolutionary analysis

#### 3.6.1 Multilocus sequence typing

E.coli_30 was classified as ST3579 with alleles *adk-*6, *fumC-29*, *gyrB-14*, *icd-16*, *mdh-24*, *purA-13*, and *recA-2*, while E.coli_45 fell under ST1121 with a different allele combination. Some of the strains selected from NCBI—GCA_001971765.1, GCA_000010385.1, and GCA_904799825.1—were identified as ST1011, ST156, and ST23 respectively, each with distinct allele profiles. Additionally, other strains isolated from our laboratory were screened using MLST, and three distinct STs were identified and selected for comparative analysis: E.coli_zq12 (ST10), E.coli_zq44 (ST95), and E.coli_zq56 (ST2522). By including different STs, we could capture the genetic diversity among the sampled *E. coli* strains, providing a robust basis for evolutionary and pangenome analysis. Supplementary Table S5 presents the STs of all the *E. coli* strains selected for the evolutionary study.

#### 3.6.2 Whole genome phylogeny

**Figure 4:**
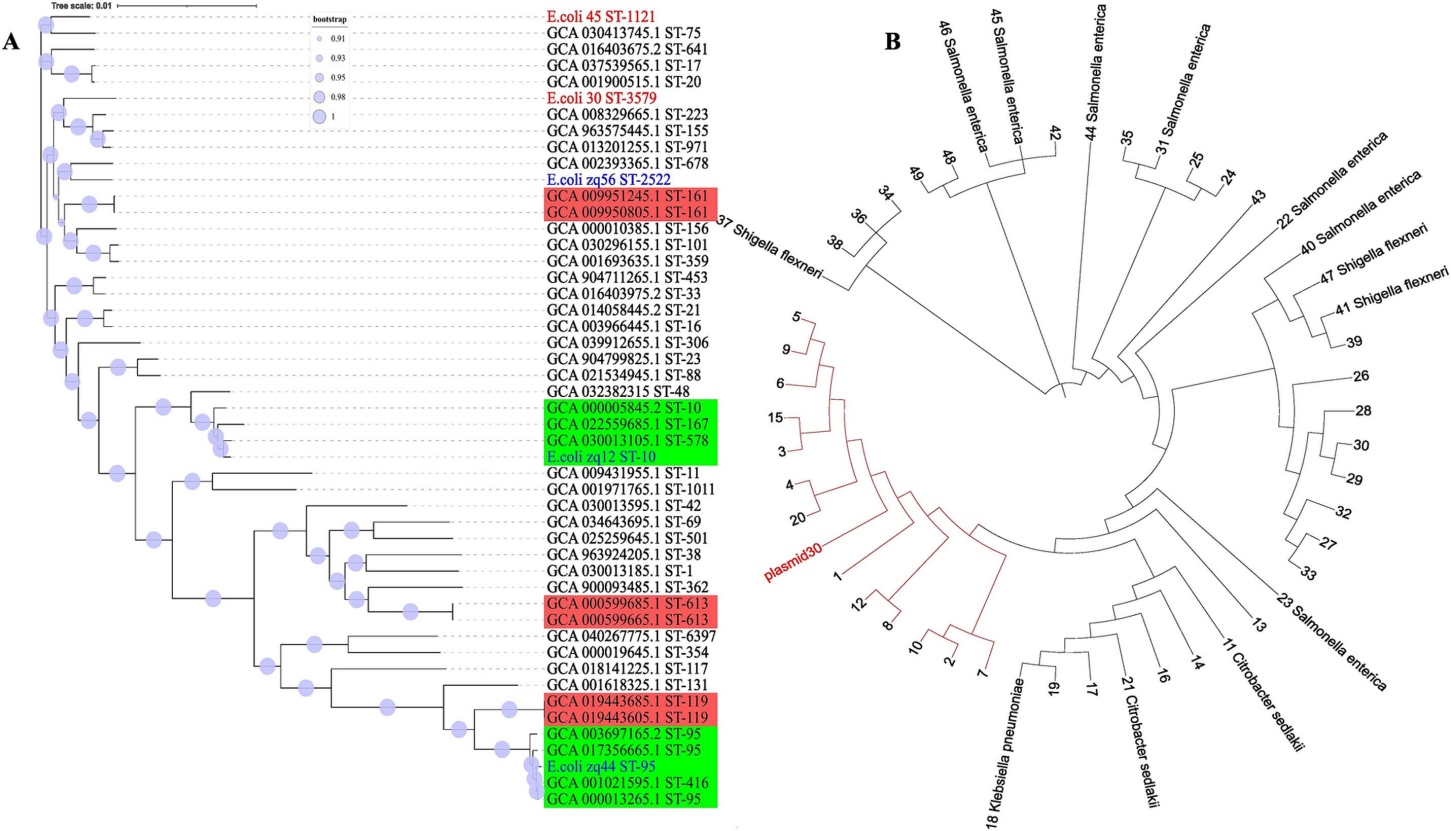
Whole genome phylogeny. **(A)** The evolutionary tree was inferred using core genomic data. Our isolates are labeled in red, those obtained from our laboratory are labeled in blue, and isolates from NCBI are labeled in black. The red clades consists of isolates with similar STs, while the green clade contains isolates with more than one ST. **(B)** Plasmid30 phylogenetic tree was constructed based on nucleotide BLAST similarity. All tips labeled with numbers represent *E. coli* isolates. The red clade includes *E. coli* plasmids that are most closely related to Plasmid30.

Evolutionary relationship among the *E. coli* genomes was inferred using sequence alignment of all 2,755 conserved genes, generated with mafft (Katoh and Standley, 2013). The phylogenetic tree (Figure 4) has Shimodaira-Hasegawa (SH) support values ranging between 0.9 and 1.0, with most clades having SH support values of 1. This indicates that our clade groupings were robust and well supported by the data. The branch lengths in the evolutionary tree represent the level of similarity or divergence; long branches suggest genetic divergence, while short branches indicate similarity or a low level of genetic diversity. The tree scale is 0.01, representing the amount of nucleotide substitution per site.

The two genomes from this study, E.coli_30 (ST3579) and E.coli_45 (ST1121), are positioned in different clades of the phylogenetic tree, indicating that they are not closely related and have distinct evolutionary lineages. The significant genetic divergence observed between them suggests that these isolates have undergone different evolutionary pressures or might have originated from different ancestral groups.

We also observed that isolates with the same STs tend to clade together, with near-zero branch lengths in such cases, suggesting minimal genetic diversity. However, some strains with the same ST showed variation and clustered with different STs. This outcome was expected, as MLST only considers the allele combinations of six housekeeping genes, while our phylogeny was based on the concatenation of over 2,700 genes. Genetic variation can occur outside the six alleles analyzed using MLST, and this explains the observed discrepancies.

For the plasmid DNA, the top 50 plasmid DNA sequences were selected using BLASTN 2.16.0+ (Zhang et al., 2000) to infer relatedness based on similarity. The clade highlighted in red (Figure 4B) consist of *E. coli* plasmids that share the closest similarity to Plasmid30. A key observation here is that plasmids from different species appeared in the top 50 BLAST hits, suggesting a high degree of similarity with Plasmid30.

#### 3.6.3 Pangenome analysis

Roary pangenome analysis revealed the distribution of genes among the 50 different *E. coli* strains we analyzed. This analysis uncovered significant divergence in gene presence across the isolates, reflecting a dynamic genome structure. We observed a reduction in the core genome size, accompanied by an expansion of the pangenome. This indicates increasing genetic diversity and the presence of strain-specific genes within the different *E. coli* STs studied.

**Figure 5:**
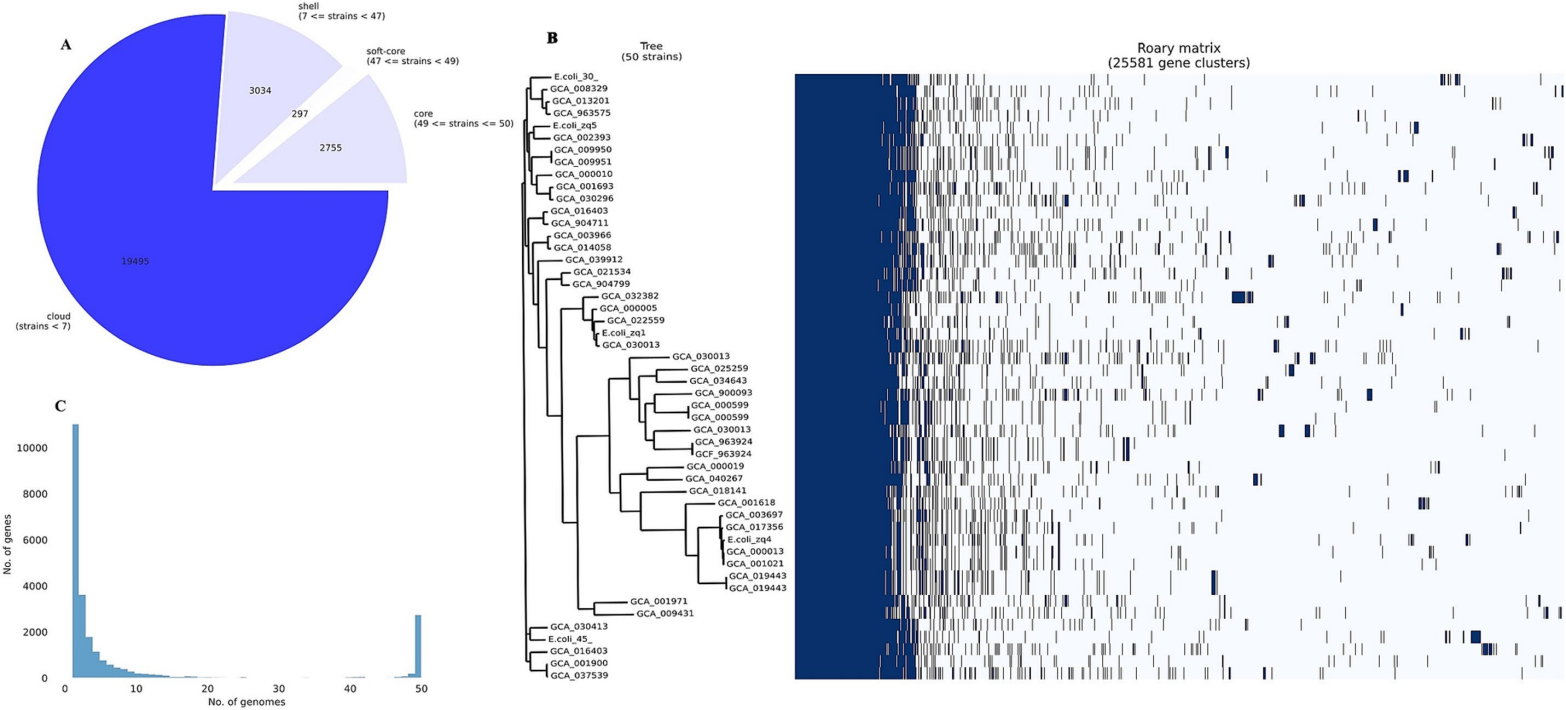
Pangenome analysis. **(A)** Pangenome pie chart showing the distribution of genes among the 50 *E. coli* strains analyzed. **(B)** Pangenome histogram showing the distribution of genes across 50 *E. coli* genomes. **(C)** Core genome phylogenetic (left) and roary matrix (right), showing the presence or absence of 25,581 gene clusters across all the 50 strains we examined.

We identified 19,495 genes in fewer than seven strains (cloud genes), 3,034 genes present in seven to fewer than 47 strains (shell genes), 297 genes in 47 to fewer than 49 strains (soft-core genes), and 2,755 essential genes (core or housekeeping genes) in 49 to 50 strains (Figures 5A,C). Many cloud genes encode hypothetical proteins with no known function. However, among the known genes, we identified *bla*_TEM-1_, *bla*_CTX-M-55_, IS110 family transposase (*IS621* and *ISEc20*), tyrosine recombinase (*xerC*), prophage tail fiber assembly protein (*tfaE*), Tn3 family transposase (*ISEc63*), IS200/IS605 family transposase (*ISEc46*), ISL3 family transposase (*ISPst2*), prophage integrase (*intS*), IS21 family transposase (*IS100kyp*), among others.

We noted that most of the cloud genes were MGEs, and several were unique to our isolates. Some unique genes in E.coli_30 include Tn3 family transposase (*TnAs1*), putative DNA ligase, e14 prophage; site-specific DNA recombinase. E.coli_45 also harbors unique genes such as fertility inhibition protein, IS4 family transposase (*IS4*), putative transposase, CP4-44 prophage toxin of the CbtA-CbeA toxin-antitoxin system, and IS110 family transposase (*ISSaen1*). We identified only one common gene between the two isolates: methyl-accepting chemotaxis protein (*mcpQ*).

The sparsely distributed clusters in Figure 5B represent the cloud genes from the 50 genomes. The shell gene section contains numerous MGEs, virulence genes (e.g., *fimD, adhesin,* and *fimC*), AMR genes (e.g., *mdtM, mdtB,* and *emrE*e), and other accessory genes, contributing to *E. coli’s* genetic diversity and adaptation.

In the soft-core and core gene sections, we found genes essential for *E. coli* survival, including multidrug export protein (*acrE*), transcriptional regulatory protein (*uhpA*), and inner membrane protein (*rclC*). These core genes are normally known to form a solid block in the pangenome matrix because they were present in all the 50 genomes in the pangenome study.

One major observation from this analysis was that *E. coli* harbors both virulence and AMR genes in its core genome, and these are essential for survival. Additionally, the accessory genome, enriched with genes acquired from other bacterial species via MGEs, contributes to the bacteria’s evolution and adaptation.

#### 3.6.4 Spread of ESBL genes *bla*_TEM_ and *bla*_CTX-M_

##### 3.6.4.1 Phylogenetic analysis across diverse microbial species

**Figure 6:**
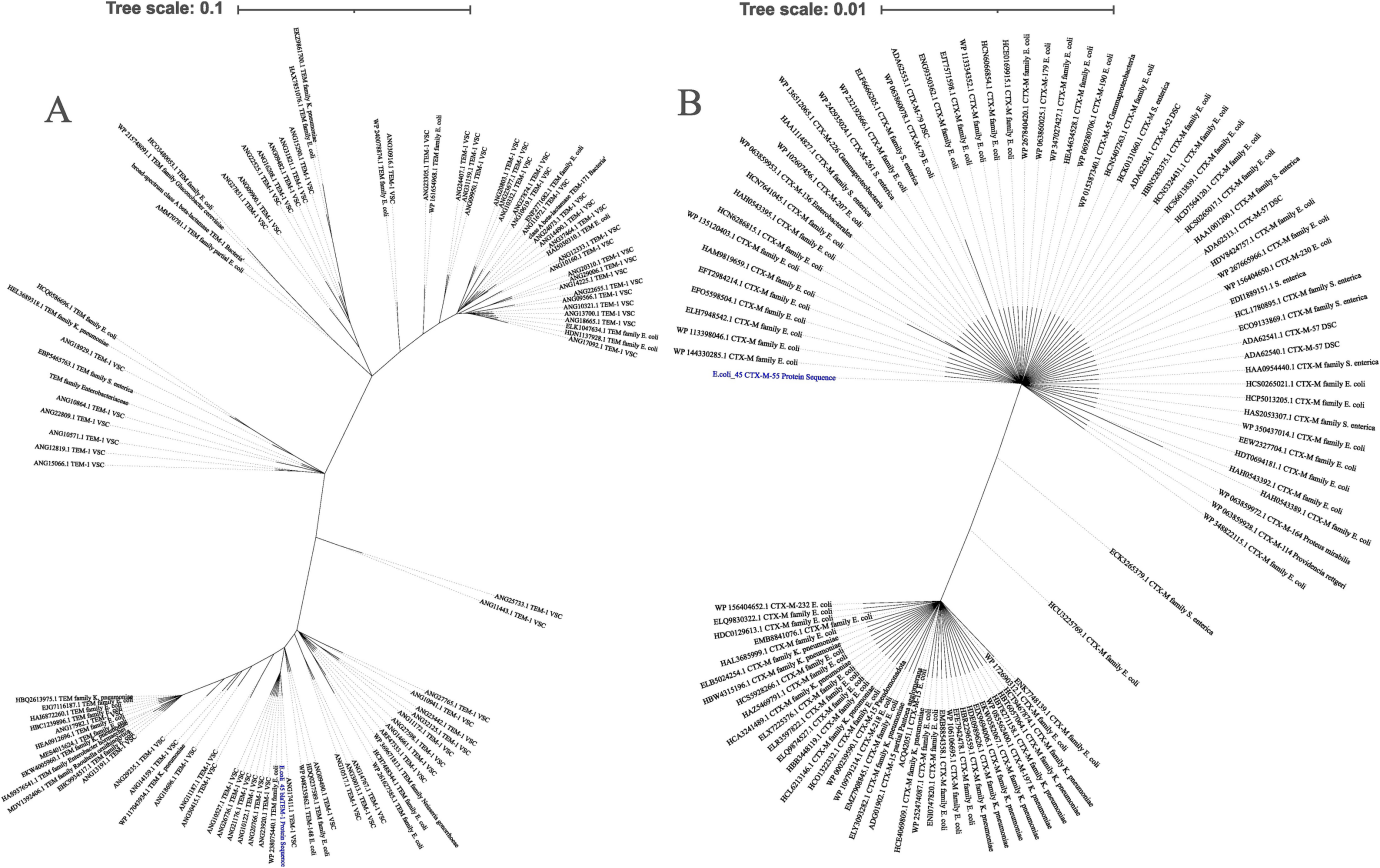
Distribution of *bla*_TEM_ and *bla*_CTM-M_ genes based on top 100 NCBI protein BLAST. VSC, variant synthetic construct; DSC, derivative synthetic construct; *K. pneomoniae*, *Klebsiella pneumoniae*; *S. enterica*, *Salmonella enterica*. **(A)** Global spread of *bla*_TEM-1_ genes inferred using maximum likelihood. **(B)** Maximum likelihood tree showing the global spread of *bla*_CTX-M_ gene family.

We studied the evolutionary distribution of the two ESBL genes identified in E.coli_45 using NCBI protein BLAST (Figure 6). The top 100 protein sequences with the highest similarity to our isolates were retrieved for comparative analysis. The ML phylogeny of *bla*_TEM_ provided insights into the similarities and differences between the E.coli_45 *bla*_TEM-1_ protein and those from public database. The gene is not highly conserved, as 41 amino acid SNPs were detected, and several variants with long branch lengths were observed. Other members of the *bla*_TEM_ gene family exist, but none were present in this phylogeny. This study uncovered that *bla*_TEM-1_ is distributed across multiple bacterial strains, with *Salmonella enterica*, *Klebsiella pneumoniae*, and *E. coli* being prevalent.

A similar analysis was conducted for the second ESBL gene (*bla*_CTX-M-55_) to examine its distribution using BLASTp. Like *bla*_TEM_, this gene is widely distributed across several microbial species, including *Salmonella enterica*, *Klebsiella pneumoniae*, and *E. coli*. Unlike *bla*_TEM_, variants of the *bla*_CTX-M_ gene family were observed in the phylogeny, such as CTX-M-15, CTX-M-57, CTX-M-190, and CTX-M-261. More amino acid SNPs (66) were detected in the *bla*_CTX-M_ comparative analysis, as more variants appeared among the top BLAST hits.

##### 3.6.4.2 Phylogenetic analysis within China

**Figure 7:**
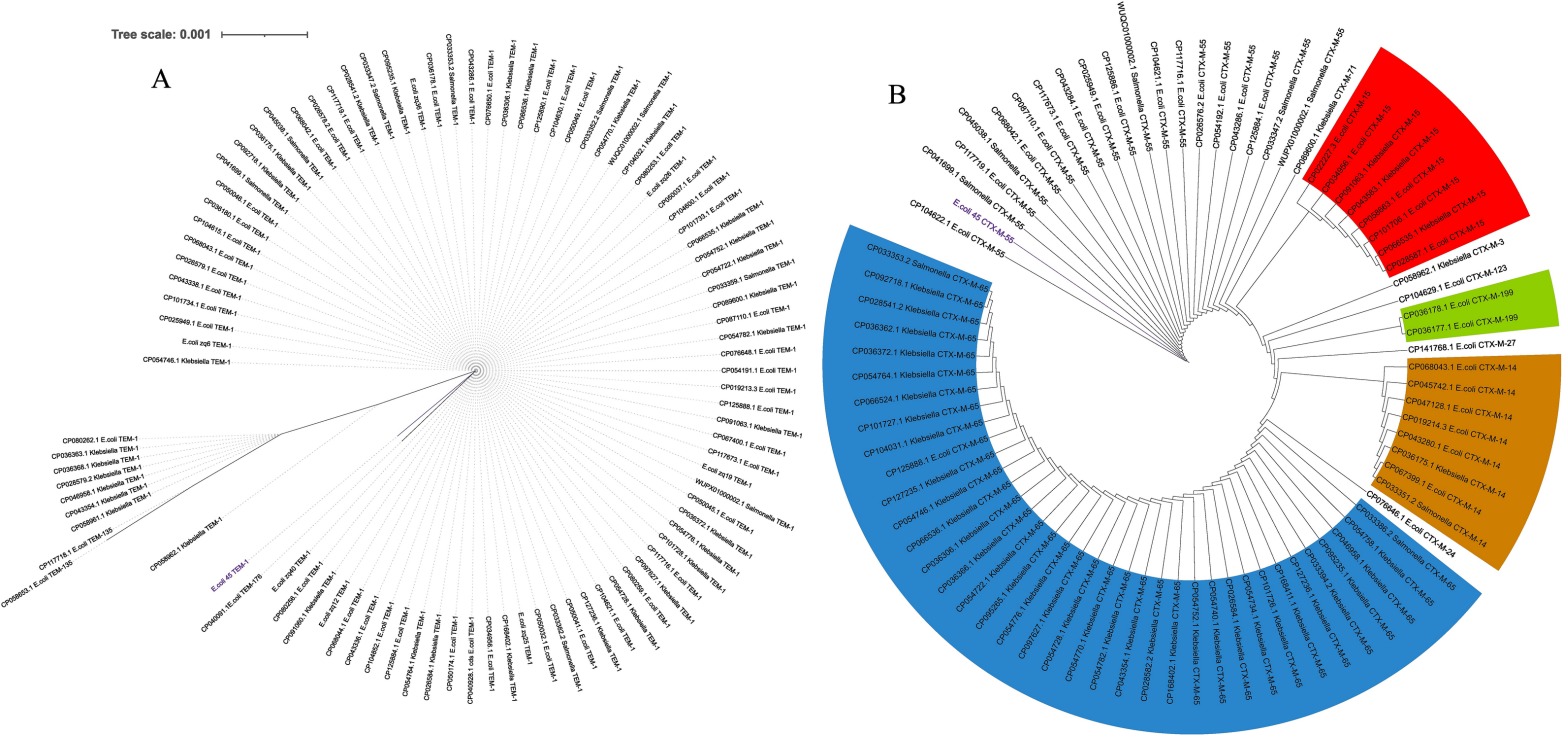
Spread of *bla*_TEM_ and *bla*_CTM-M_ genes within China across three bacterial species from 2019 to 2024. **(A)** Maximum likelihood tree of *bla*_TEM_ genes. **(B)** Maximum likelihood tree of *bla*_CTX-M_ gene family.

An additional evolutionary analysis was conducted to investigate the spread of these genes within China. This study focused on *E. coli, Salmonella enterica*, and *Klebsiella pneumoniae*, as our previous analysis (Figure 6) indicated that these genes were most prevalent in these three species. We examined the presence of these genes directly from the genomes of hundreds of isolates from China over the past 5 years. For the *bla*_TEM-1_ gene (Figure 7A), we detected it 92 times across *E. coli*, *Salmonella enterica*, and *Klebsiella pneumoniae*, with two additional variants, TEM-135 and TEM-176, also identified. Multiple occurrences of the gene within single genomes were observed, suggesting its spread through MGEs. Although other family members were detected, TEM-1 was the most prevalent, and the gene appeared highly conserved across the three bacterial species. We noted the presence of an SNP in the E.coli_45 *bla*_TEM-1_ gene and this explains its significant branch distance in the dendrogram. The gene was completely conserved across all *Salmonella strains* and several *Klebsiella* and *E. coli* genomes, including six strains (E.coli_zq6, E.coli_zq12, E.coli_zq40, E.coli_zq25, E.coli_zq19, and E.coli_zq26) isolated in our laboratory. The longest branch length was found in the two *bla*_TEM-135_ variants, which clustered at the bottom of the tree, showing the most genetic diversity.

We performed a similar analysis to investigate the distribution of *bla*_CTX-M_ across China (Figure 7B). Comparative analysis between the three species revealed that *bla*_CTX-M-55_ was predominantly found in *E. coli* and *Salmonella*. Each gene within the *bla*_CTX-M_ family formed distinct clusters, regardless of the host species. Several variants were detected, including CTX-M-15, CTX-M-65 (prevalent in *Klebsiella*), CTX-M-14, and CTX-M-199. These genes were found multiple times within a single genome, and this suggests their spread via MGEs. Another key observation was that *bla*_CTX-M-55_ clustered near the root of the tree. This suggests that it was the ancestral variant of the other *bla*_CTX-M_ variants.

### 3.7 Detection of mobile genetic elements

Several MGEs were identified in the two MDR *E. coli* genomes. MGEfinder was used to detect several insertion sequences and their insertion sites in the two genomes. Some insertions were very short sequences, approximately 20 bp, while others were larger, ranging from 1,000 to 3,000 bp. Annotation of the larger regions revealed the presence of IS110 family transposases, IS3, and several passenger genes, including *invasin, electron transfer flavoprotein-ubiquinone oxidoreductase, ygcP, and fixB*, among others, in E.coli_45. We also found IS110 and IS3 transposases in E.coli_30, with passenger genes including *rhm1*, *algC*, and *btuD*. We also found 20 inserted genes in one larger insertion site in E.coli_30.

**Figure 8:**
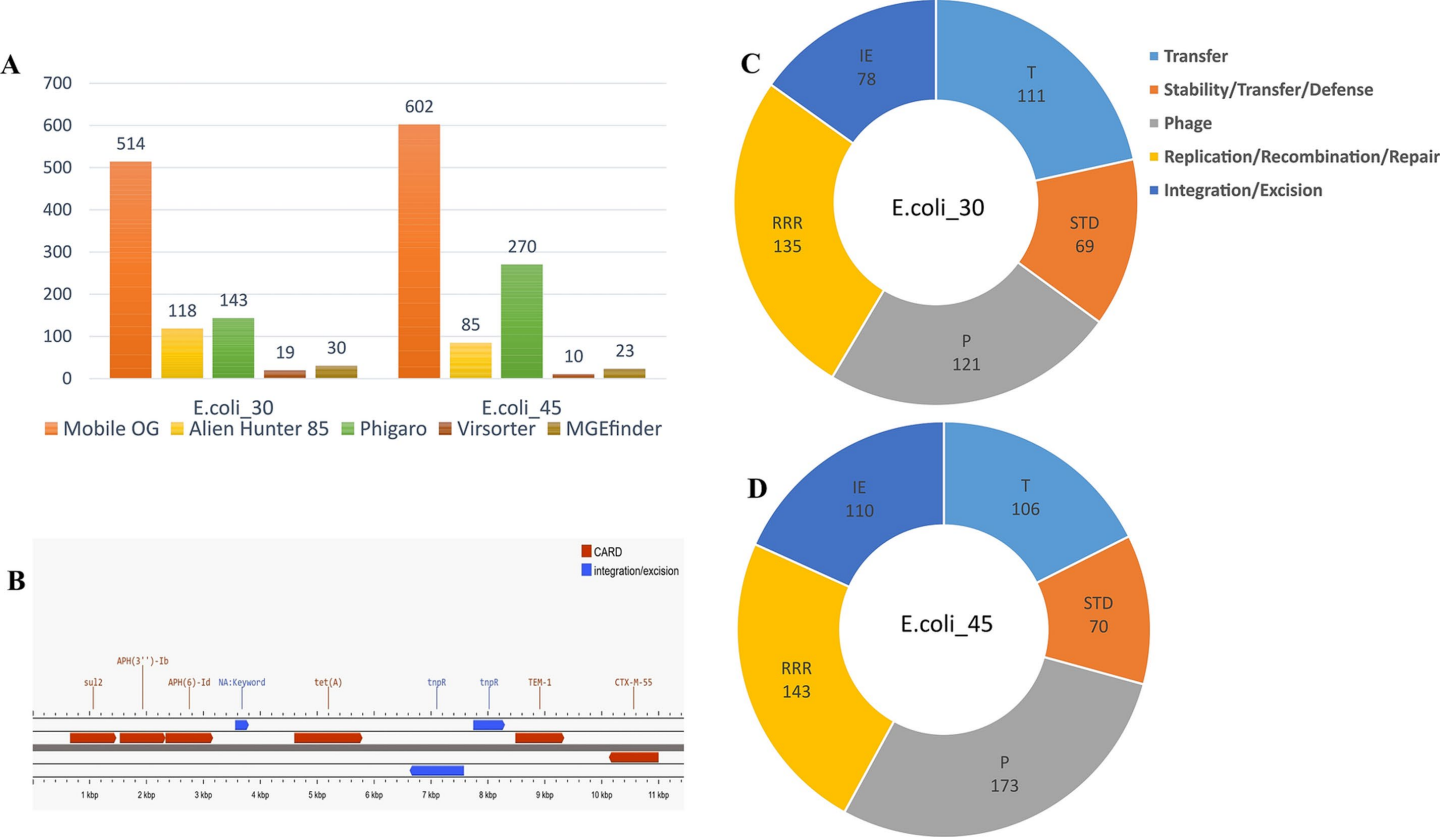
Mobile genetic elements. **(A)** Mobile elements hits identified using different tools. **(B)** Annotation of the contig (locus) carrying ESBL genes in E.coli_45. **(C)** MGEs hits in E.coli_30 using MobileOG-db. **(D)** MGE hits in E.coli_45 using MobileOG-db.

Furthermore, the genomes were then annotated using MobileOG-db, which tracks mobile elements by organizing them into orthologous groups. We tracked a wide range of MGEs, including transposons, integrons, IS elements, phage related genes, and integrative conjugative elements, which explains the large number of MGEs found within the two genomes (Figure 8A). Among the identified MGEs, we found genes involved in critical processes such as replication, recombination, repair, integration/excision, and phage-related functions (Figures 8C,D). Notable genes include *sdiA*, associated with conjugation and relaxosome activity, and *ruvA*, *ruvC*, and *holE_2*, which play key roles in DNA repair, recombination, genome stability, and horizontal gene transfer (HGT). Additionally, phage-related genes such as *dicB*, *essD*, and *quuQ_2* that contribute to lysis, lysogeny, and infection regulation, were detected. Transposon-associated genes such as *tnpA* and *tnpB* facilitate integration and excision, while stability/transfer/defense genes like *hipA* and *cnu* help maintain genetic integrity. We observed that some entries for both genomes were manually curated, while others were annotated through automated homology-based methods using several databases.

The phage genome annotator PhiGARo predicted prophages and other virus-like sequences in conjunction with virus orthologous groups (VOGs). We identified five prophage regions and 138 prophage genes in E.coli_30. In the second genome, we predicted 11 prophage regions and 259 genes, significantly more than those in E.coli_30. The taxonomy of these prophage genes revealed that most belonged to the *Myoviridae* family, a few were of unknown origin, and one was classified as *Podoviridae*. The prophage genes identified were associated with roles such as lysis, integration, termination, tail assembly, replication, coat formation, and portal functions. HGT regions and their insertion sites within the two genomes were predicted using Alien Hunter, while viral signals were detected using VirSorter within the two genomic sequences (Figure 8A).

Finally, we further investigated the locus harboring the ESBL genes in E.coli_45 and found that the *bla*_TEM-1_ gene was likely inserted into the genome via the Tn3 insertion sequence. Annotation of this locus revealed a Tn3 family transposase (*Tn2*) and a site-specific DNA recombinase *(tnpR)* located just a few bases upstream of the ESBL gene (Figure 8B), which likely facilitated its integration. The closest match we found using ISfinder BLAST for this locus showed the presence of a DDE recombinase (IS110 family transposase), Tn3 resolvase, and a passenger gene (*bla*_TEM-1_), and this result aligns closely with our findings.

## 4 Discussion

### 4.1 Phenotype resistance profile

The findings from the antibiotic resistance profiles among the *E. coli* isolates provided vital insights into the response of each antibiotic against the tested bacterial isolates. In this study, GEN exhibited the most efficacy against the *E. coli* isolates tested, while IMP and CIP, on the other hand, exhibited high levels of resistance. Shams et al. (2018) documented high levels of carbapenem-resistant *E. coli*, while Shariati et al. (2022) reported CIP resistance in the context of urinary tract infections and other *E. coli*-associated diseases.

Interestingly, the susceptibility test showed that our isolates displayed much greater levels of resistance to IMP compared to AMP, a first-generation penicillin. This situation could have occurred due to several reasons. One possibility is that when certain antibiotics begin to show reduced efficacy against bacteria, their increased consumption may result in heightened resistance. This explains why IMP-resistant bacteria are often associated with carbapenem intake, possibly corroborating our findings (Yang et al., 2018).

Another plausible reason is that the IMP-resistance isolates in our study may habor carbapenem-specific *β*-lactamases such as New Delhi Metallo-β-lactamase (NDM) or *Klebsiella pneumoniae* Carbapenemase (KPC) without ESBLs or AmpC enzymes, thus remaining susceptibility to AMP. For instance, Mojica et al. (2022) reported that some *E. coli* strains harboring NDM remain susceptible to some penicillins due to the specific substrate profile of NDM enzymes.

Additionally, IMP resistance could be due to alterations (mutations) in porin channels that limit the entry of IMP without affecting AMP. This phenomenon has been reported in clinical isolates where specific mutations in porin proteins confer carbapenem resistance (Poirel et al., 2020). Furthermore, efflux pumps, such as those of the RND family, which confer MDR, may have substrate specificity that includes IMP but not AMP, potentially contributing to the resistance patterns observed (Tambat et al., 2022).

### 4.2 Antimicrobial resistance genes

The identification and characterization of AMR genes in our *E. coli* isolates revealed several key resistance mechanisms. The presence of pmr phosphoethanolamine transferases (e.g., *marA* and *acrD*), efflux pumps (e.g., *acr*AB-tolC), and β-lactamase indicate that these isolates possess vast mechanisms to evade antibiotic treatment. These findings agree with other studies that have reported the widespread presence of AMR genes in some *E. coli* strains, posing major problems to public health (Yang et al., 2018; Majumder et al., 2021). Efflux pumps play a crucial role in mediating bacterial resistance by expelling antibiotics from the cells. To combat this, Lu et al. (2024) developed a potential efflux pump inhibitor (EPI) derived from a seaweed compound, which displayed efficacy in reducing AMR in drug-resistant *E. coli* strains. Another study explored the use of a microbe-derived EPI, ethyl 4-bromopyrrole-2-carboxylate (RP1), which shows promise in combination therapies (Tambat et al., 2022). The findings from that study indicate significant reductions in minimum inhibitory concentrations and extended post-antibiotic effect, making RPI a promising candidate for enhancing existing antibiotic treatments.

We noted the presence of specific *β*-lactamase genes (*bla*_TEM-1,_ *bla*_CTX-M-55_) in E.coli_45 (Figure 8B), making this isolate an ESBL-producing *E. coli* (Chen et al., 2019; Papouskova et al., 2020). The presence of ESBL enzyme in E.coli_45 indicates that this strain is resistant to most β-lactam antibiotics, posing a significant public health risk due to limited treatment options (Chen et al., 2019). A recent pharmaceutical strategy to address this issue involves the use of β-lactamase inhibitors. These inhibitors are crucial in restoring the activity of β-lactam antibiotics against bacteria that produce β-lactamase enzymes. Le Terrier et al. (2024) studied taniborbactam, a novel broad-spectrum β-lactamase inhibitor effective against metallo-β-lactamases (MBLs). Their study showed that taniborbactam exhibited significant activity against B1 subclass MBLs, including NDM and VIM (Verona integrin-encoded metallo-β-lactamase) enzymes (Le Terrier et al., 2024). However, certain variants such as NDM-9 and VIM-83 demonstrated resistance, raising concerns regarding the potential emergence of resistant strains (Le Terrier et al., 2024). Taniborbactam’s ability to inhibit a wide range of MBLs, including those resistant to traditional β-lactamase inhibitors, positions it as a critical tool in the fight against MDR pathogens.

In addition to β-lactam resistance, several other AMR genes were detected in both isolates. This underscores the critical need for monitoring and developing novel therapeutic strategies, such as bacteriophage therapy, to combat MDR (Montso et al., 2019). We also identified multiple AMR genes associated with resistance to various classes of antibiotics, including quinolones (*qnr* genes), sulfonamides (*sul* genes), and tetracyclines (*tet* genes). The presence of numerous variants of these genes within the MDR isolates suggests HGT as a major facilitator of AMR (Partridge et al., 2018). This finding aligns with research that highlights the role of plasmids, integrons, and transposable elements, such as IS, in spreading AMR across *E. coli* and other bacterial species (Zingali et al., 2020; Vinayamohan et al., 2022).

### 4.3 Virulence associated genes

The analysis of virulence-associated genes in the test isolates revealed the presence of genes associated with adhesion (e.g., *cfaB* and *fimH*), invasion (e.g., *ibeC* and *ompA*), effector delivery systems (TTSS effectors), and metabolic factors or iron acquisition (e.g., siderophores like enterobactin and salmochelin) (Algammal et al., 2020). Notably, salmochelin siderophore virulence genes, such as *iroB* and *iroC,* were exclusively found in E.coli_30 and its plasmid. These virulence identifiers (adhesion, invasion, and nutrition) are vital for the pathogenic potential of *E. coli*, enabling the bacterium to colonize hosts, evade immune responses, and cause disease (Croxen et al., 2013).

Our findings are supported by previous research highlighting the wide range of virulence-associated genes found in pathogenic *E. coli* isolates, which significantly contribute to the bacteria’s ability to cause different types of infections (Sarowska et al., 2019; Sora et al., 2021). The identification of virulence genes, such as the TTSS effectors (e.g., *espX1, espX4,* and *espL1*) and iron uptake systems like salmochelin, in our isolates suggests their potential to cause severe infections (Radics et al., 2014; Magistro et al., 2015). These identified factors, including invasin, adhesin, and metabolic factors, facilitate bacterial invasion, intracellular survival, and nutrient acquisition, respectively, which are essential for causing infections in a host organism (Liu et al., 2022).

The presence of virulence genes in both commensal and pathogenic bacterial strains has been reported and these genes underscore the complex relationship between *E. coli* and its host, determining the outcome of infections (Dionisio et al., 2023).

### 4.4 Phylogenetic relationships and evolutionary insights

The phylogenetic tree generated in this study revealed distinct clades, each representing different evolutionary lineages. The isolates we sequenced, E.coli_30 (ST3579) and E.coli_45 (ST1121), were placed in different clades, highlighting a major genetic divergence and suggesting that these two isolates have been through different evolutionary pressures or they may have come from different ancestral populations. What we observed here agreed with previous studies that reported on the genetic diversity within *E. coli* strains driven by various evolutionary factors such as mutations, HGT via MGEs, and genetic recombination (Vinayamohan et al., 2022; Touchon et al., 2009). The diverse placement of isolates with different STs in the phylogenetic tree indicate a wide genetic diversity even within smaller groups of *E. coli* strains. This aligned with the result of a study by Didelot et al. (2012) in which they reported on the diversity of *E. coli* with dynamic adaptation to many hosts and environmental conditions, leading to major genome plasticity. The 45 isolates from the NCBI database were distributed across various clades in the phylogeny, except for isolates with the same STs (mostly tend to clade together). This highlights the extensive genetic diversity present in the NCBI database for *E. coli* strains. The varied clustering patterns we observed in the phylogenetic tree suggest that these *E. coli* isolates were arranged into multiple sub-types with each strain following its unique evolutionary paths (Touchon et al., 2020).

The phylogenetic analyses of *bla*_TEM-1_ and *bla*_CTX-M_ genes in *E. coli*, *Salmonella enterica*, and *Klebsiella pneumoniae* from China showed how widely these resistance genes have spread, thanks to MGEs. The discovery of multiple copies of these genes within individual genomes and across species highlights the important role of MGEs, such as IS and transposons, in moving these genes between bacteria. *bla*_TEM-1_ appeared to be highly conserved, meaning it has changed little across species, while *bla*_CTX-M_ variants, such as CTX-M-55 and CTX-M-15, showed more diversity, forming distinct clusters that reflect different evolutionary paths. This mirrors the findings from other studies that show plasmids helping to spread resistance genes across bacterial species in China (Wu et al., 2015; Kuang et al., 2018). The presence of small mutations, or SNPs, in some of the genes suggests that they are continuing to evolve. Overall, these results emphasize how MGEs drive the spread of antibiotic resistance across bacteria, agreeing with global trends observed in similar studies (Peng et al., 2024; Jin et al., 2015; Awosile and Agbaje, 2021).

### 4.5 Pangenome analysis

The pangenome analysis conducted in this study included both core and accessory genomes to elaborate extensively on the genetic diversity within 50 *E. coli* strains. The core genomes has conserved genes that are present in all the isolates, shedding knowledge on essential functions and evolutionary conserved mechanisms. The accessory genome on the other hand, consisting genes present in some but not all isolatesprovided insights into the adaptive potential of *E. coli* to many different environments and antibiotic treatments (Vernikos et al., 2015). The results of the pangenome analysis revealed a high proportion of unique genes in individual isolates, indicating strain-specific *E. coli* adaptations. Recent studies on *E. coli* pangenome (gene-specific) support what we reported that new genes continue to be obtained through HGT, helping in the bacteria’s adaptation and vast ecology (Hall et al., 2021). The identification of genes associated with metabolism (iron uptake), stress response, and adhesion, highlights the adaptive nature of the *E. coli* genome, facilitating its survival in diverse environments (Liu et al., 2022).

### 4.6 Mobile genetic elements

Our investigation into MGEs across the two genomes revealed that the results varied significantly depending on the analytical tools used. Each tool offered distinct methodologies for MGE identification and these helped illuminate different aspects of MGE diversity and behavior. MGEfinder emerged as the most reliable tool in this study, providing detailed mapping of insertion sites and accurately identifying associated insertion sequences. This tool successfully recognized IS elements, whether or not they contained inverted terminal repeats, which are essential features for many MGEs. In particular, sequences with terminal repeats, such as those from the IS3 family transposase (IS3) and ISEc17 were detected in the two genomes and across several other genomes when studying the spread of ESBL genes. These elements are widespread in *E. coli* genomes and often carry critical passenger genes such as *bla*_TEM-1_ and *bla*_CTX-M_ that contribute to antibiotic resistance. This finding aligned with prior studies that emphasized the role of IS elements in disseminating resistance genes (Partridge et al., 2018). Additionally, MGEfinder was able to identify elements that lacked inverted terminal repeats, which are predominantly part of the IS110 family (Durrant et al., 2024). In both the two genomes, we noted the presence of IS621, an essential member of the IS110 recombinase. The integration mechanism for this family involves a recently discovered bridge RNA recombination process (Durrant et al., 2024; Hiraizumi et al., 2024), and this underscores a novel pathway for MGE mobility.

Complementary to MGEfinder, other tools such as MobileOG-db provided MGE characterization by annotating the genomes against a variety of databases. MobileOG-db matched sequences with several key databases, including pVOG, Plasmid RefSeq, GPD, and ICE. This broadened the scope of our analysis and yielded a large number of hits for MGEs in our isolate. In addition, we employed VirSorter, PhiGARo, and Alien Hunter, which further contributed to MGE detection across the genomes by targeting specific MGE types. VirSorter has been widely used in recent virome studies (Roux et al., 2015) to identify viral sequences embedded in bacterial genomes, a feature relevant to our study given the potential role of viruses in HGT. Similarly, PhiGARo and Alien Hunter have previously demonstrated effectiveness in detecting prophages and alien DNA, respectively, complementing each other by expanding the types of MGEs detected (Akhter et al., 2012).

## Data Availability

The data presented in the study are deposited in the NCBI repository, BioProject accession number PRJNA1169417.

## Footnotes

^1^ https://card.mcmaster.ca/
