# Supplementary material for: Genomic analysis of multidrug-resistant Escherichia coli isolated from dairy cows in Shihezi city, Xinjiang, China
Source: Front Microbiol. 2025 Feb 26;16:1527546. doi: 10.3389/fmicb.2025.1527546 (PMC11934113; doi:10.3389/fmicb.2025.1527546)
Supplement: Supplementary file 6 [file Table_6.DOCX]

Supplementary Material

# Supplementary Figures and Tables

## Supplementary Tables

**Supplementary Table 1.** E.coli_30 antimicrobial resistance ontology

**Supplementary Table 2.** E.coli_45 antimicrobial resistance ontology

**Supplementary Table 3.** E.coli_30 virulence factor

**Supplementary Table 4.** E.coli_45 virulence factor

**Supplementary Table 5.** Multilocus sequence typing
